# The Prognostic Value of the Chromobox Family in Human Ovarian Cancer

**DOI:** 10.7150/jca.44475

**Published:** 2020-07-06

**Authors:** Yichi Xu, Shuya Pan, Yizuo Song, Chunyu Pan, Cheng Chen, Xueqiong Zhu

**Affiliations:** Department of Obstetrics and Gynecology, The Second Affiliated Hospital of Wenzhou Medical University, Wenzhou, China.

**Keywords:** ovarian cancer, Chromobox (CBX), immunohistochemistry (IHC), survival analysis, prognosis

## Abstract

Ovarian cancer is one of the most lethal gynecologic tumors in women and has a poor prognosis. The purpose of our study was to identify new prognostic markers in ovarian cancer. We examined the prognostic roles of mRNA expression of the chromobox (CBX) family in patients with ovarian cancer utilizing the Kaplan-Meier plotter database. The prognostic values and expression levels of CBX members associated with prognosis were further evaluated using KM plotter in diverse subgroups and immunohistochemistry (IHC) analysis in ovarian carcinoma. The results revealed that elevated CBX1-3 mRNA expression may predict poor overall survival (OS) and progression-free survival (PFS) outcomes in patients with ovarian cancer. Notably, in women with ovarian cancer, increased CBX1 mRNA expression was linked to a short OS in all stages and in the grade II and grade III subgroups. Additionally, CBX2 and CBX3 were strongly related to short OS in stage III+IV patients, and a link between high CBX3 mRNA expression and unfavorable OS in grade II patients was observed. High expression levels of CBX1 and CBX3 were significantly associated with chemotherapy resistance in ovarian cancer patients. IHC staining showed that the CBX1-3 proteins were upregulated in serous ovarian carcinoma tissues compared with normal ovarian tissues. Therefore, our results indicated that CBX1-3 could be attractive biomarkers for predicting poor prognosis of ovarian cancer.

## Introduction

Ovarian cancer is one of the most prevalent gynecologic malignancies worldwide. According to statistics, the number of ovarian cancer-related deaths reached 13980 in 2019 in the United States 1. Platinum-based chemotherapy and cytoreductive surgery are the standard treatments for patients with advanced malignant ovarian tumors 2, 3. Nonetheless, most of these patients ultimately develop recurrence and chemoresistance with a dismal 5 year overall survival less than 50% due to the predominance of aggressive late stage ovarian carcinomas and a lack of models with high sensitivity for predicting prognosis 4, 5. Therefore, identification of a novel prognostic biomarker is important for prediction of progression, recurrence and the overall therapeutic effect as well as for improving the clinical outcomes of patients with this deadly disease.

The chromobox (CBX) members are the crucial components of polycomb repressive complexes 1 (PRC1), which are critically involved in the regulation of various biological functions such as gene expression and body development 6. CBX proteins are composed of eight members that are further divided into two groups: (1) the polycomb CBX proteins modulate transcription of target genes via interaction with the core PRC1 complex, including CBX2, CBX4, CBX6, CBX7, and CBX8 7, 8, and (2) heterochromatin protein 1 (HP1) is essential for gene silencing, DNA repair, and telomere function and contains CBX1 (also known as HP1β), CBX3 (HP1γ), and CBX5 (HP1α) 9, 10.

Accumulated evidence has indicated the dual role of the CBX family in cancer development, as the members can serve as both tumor suppressors and oncoproteins 6. For example, the CBX3 protein is commonly upregulated in human colorectal cancer and promotes cancer cell proliferation both *in vitro* and *in vivo* 11. Immunohistochemistry-based clinicopathological studies showed that the expression of the CBX1 protein was significantly elevated in human hepatocellular carcinoma (HCC) specimens 12, and CBX2 protein expression was elevated in human prostate cancer tissues 13, predicting a poor outcome in these patients. Conversely, Wang *et al.* revealed that the CBX8 protein inhibits the metastasis of esophageal squamous cell carcinoma by suppressing Snail expression 14. Specifically, the CBX7 protein has been found to be abundantly expressed in hematopoietic stem cells whereas the upregulation of CBX7 induced self-renewal of stem cells and leukemia formation 15. However, contrasting studies showed that CBX7 expression was dramatically decreased in cancer cells 16, 17 and that *CBX7* knockout (*CBX7^-/-^*) mice tended to develop liver and lung carcinomas 17.

However, the roles of CBX family genes in carcinogenesis and progression of ovarian cancer have not been fully elucidated. Hence, in the current study, computer data mining methods were employed to uncover the prognostic value of the CBX family in ovarian cancer. In addition, IHC analysis was used to elucidate the differential expression of CBX members correlated with prognosis in normal ovarian and ovarian cancer tissues. This study suggested that some CBX members might be considered attractive prognostic indicators in ovarian cancer in the future.

## Materials and Methods

### Kaplan-Meier plotter

The online Kaplan-Meier plotter database (http://kmplot.com/analysis/) 18 was utilized to determine the correlation of the mRNA expression of individual CBX members and survival outcomes (OS and PFS) in patients with ovarian cancer. The background database could evaluate the influences of 54,675 genes on the survival rates for breast cancer 18, lung cancer 19, gastric cancer and ovarian cancer 20. Currently, a total of 1287 patients with ovarian cancer were analyzed in the European Genome-Phenome Archive, the Cancer Biomedical Informatics Grid, the Gene Expression Omnibus and The Cancer Genome Atlas cancer datasets 20. In addition, several clinicopathological parameters such as histology, pathological grade, and clinical stage, as well as applied chemotherapy for ovarian cancer patients were obtained from this database.

Then eight CBX subtypes (CBX1, CBX2, CBX3, CBX4, CBX5, CBX6, CBX7 and CBX8) were entered into the Kaplan-Meier plotter database (http://kmplot.com/analysis/index.php?p=service&cancer=ovar) to download Kaplan-Meier survival plots. To further determine the prognostic value of a specific candidate molecule, we assigned these samples to two groups. CBX mRNA expression status was classified as 'high' and 'low' in accordance with the gene expression values with preestablished cutoffs. The two patient cohorts were compared to the Kaplan-Meier survival plots, with hazard ratios (HRs), 95% confidence intervals (95% CIs) and log-rank *P* values exhibited on the web page. A *P* value less than 0.05 was considered significant.

### Tissue samples

From August 2017 to December 2018, 18 samples from serous ovarian cancer in paraffin embedded slides and normal ovarian tissues were obtained from the Second Affiliated Hospital of Wenzhou Medical University. A total of 18 samples of malignant ovarian tumor tissue were taken from patients confirmed to have serous ovarian cancer by pathological diagnosis after surgery, and the median age was 50 years (range 34-65 years). There were 18 normal ovarian tissues obtained from bilateral oophorectomy due to unilateral ovarian benign lesions (control group), and the median age was 56 years (range 45-63 years).

This study was carried out in agreement with the Declaration of Helsinki and was approved by the Ethical Committee of the Second Affiliated Hospital of Wenzhou Medical University. Sample collection and analysis were performed after obtaining informed consent from each subject.

### Immunohistochemistry

The paraffin-embedded tissues were cut into 4 µm slices and subsequently incubated overnight at 65°C. After treatment with deparaffinization and rehydration, the tissue slices were heated in a microwave oven with sodium citrate solution (10 mM, pH 6.0) at 95°C for 20 min to repair the antigen. Then, the slices were allowed to cool naturally to room temperature. Next, the slices of tissue were washed twice with PBS and incubated in 3% hydroxyl peroxide for 15 min to inactivate endogenous peroxidase activity. Then, the tissues were blocked in 3% bovine serum albumin (BSA) for 40 min at room temperature. Subsequently, these slices were incubated with the primary CBX1 antibody (dilution: 1:400; ab10478, Abcam), CBX2 antibody (dilution: 1:200; ab235305, Abcam) and CBX3 antibody (dilution: 1:2000; ab217999, Abcam) at 4°C overnight. The tissue slides were incubated with the secondary antibody (anti-rabbit Dako Envision+ System HRP Labeled Polymer, Dako Ref#K4003) at room temperature for half an hour. The tissue sections were washed with PBS and subjected to antigen detection staining with 3,3'-diaminobenzidine (DAB) solution (Dako Denmark A/S, Glostrup, Denmark). The slides were stained by using DAB for 2 min and counterstained with hematoxylin to strengthen the nuclear staining. Finally, the slides were installed, dehydrated by xylene and covered. The positive and negative controls were stained in parallel. The expression of the CBX1, CBX2 and CBX3 proteins was selected for further study in ovarian cancer tissues based on both the intensity of staining and percentage of positive cells which were blindly assessed by two independent observers. The intensity of staining was graded as follows: 0 (no staining), 1 (mild staining), 2 (moderate staining) and 3 (intense staining). The percentage of positive cells was graded as follows: 0 (0-5%), 1 (6-24%), 2 (25%-49%), 3 (50%-74%) and 4 (75%-100%). The immunoreactive score was acquired by multiplying the positive cell fraction scores and the staining intensity score.

### Statistical and survival analysis

All statistical analyses were processed using SPSS statistical software (version 18.0; Chicago, IL, USA). The CBX1, CBX2 and CBX3 protein expression levels between the cancer group and the control group were compared by two-tailed Student's *t* test. Survival curves were depicted by utilizing the Kaplan-Meier method with hazard ratios (HRs), 95% confidence intervals (95% CIs) and log-rank *P* values. When the *P* value was <0.05, the differences were deemed to be statistically significant.

## Results

### Prognostic value of different CBX family genes in all ovarian cancer patients

**Figure 1:**
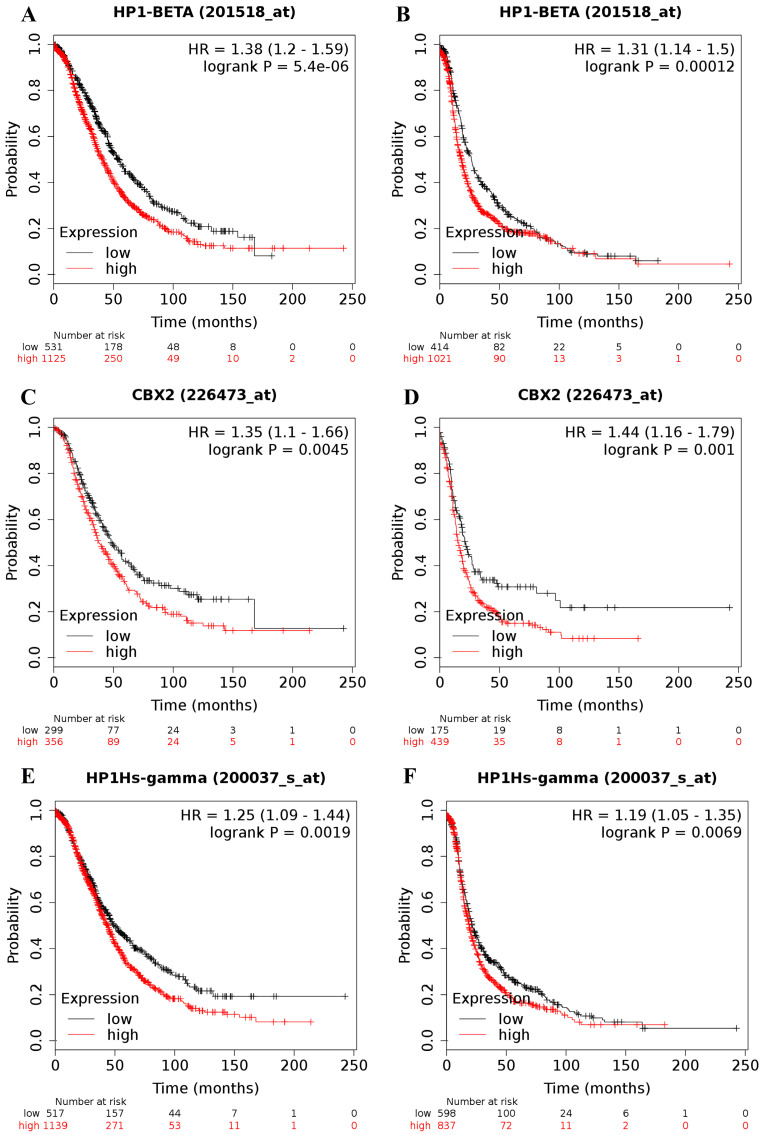
The prognostic value of CBX1-3 mRNA expression in ovarian cancer. Its Affymetrix ID is 201518_at, 226473_at and 200037_s_at.** (A)** OS curves are plotted for all ovarian cancer patients (n = 1,656). **(B)** PFS curves are plotted for all ovarian cancer patients (n = 1,435). **(C)** OS curves are plotted for all ovarian cancer patients (n = 655). **(D)** PFS curves are plotted for all ovarian cancer patients (n = 614). **(E)** OS curves are plotted for all ovarian cancer patients (n = 1,656). **(F)** PFS curves are plotted for all ovarian cancer patients (n = 1,435).

The online Kaplan-Meier plotter database was applied to explore the prognostic value of individual CBX family members at the transcriptional level in patients with ovarian cancer. Increases in CBX1 (Figure 1A and 1B), CBX2 (Figure 1C and 1D) and CBX3 (Figure 1E and 1F) at the transcriptional level were all strongly related to short OS and PFS for ovarian cancer patients (OS: CBX1: HR = 1.38 (1.20 - 1.59), *P* = 0.0000; CBX2: HR = 1.35 (1.10 - 1.66), *P* = 0.0045; CBX3: HR = 1.25 (1.09 - 1.44), *P* = 0.0019; PFS: CBX1: HR = 1.31 (1.14 - 1.50), *P* = 0.00012; CBX2: HR = 1.44 (1.16 - 1.79), *P* = 0.0010; CBX3: HR= 1.19 (1.05 - 1.35), *P* = 0.0069). The desired Affymetrix IDs for CBX1, CBX2, and CBX3 are 201518_at, 226473_at, and 200037_s_at, respectively.

**Figure 2:**
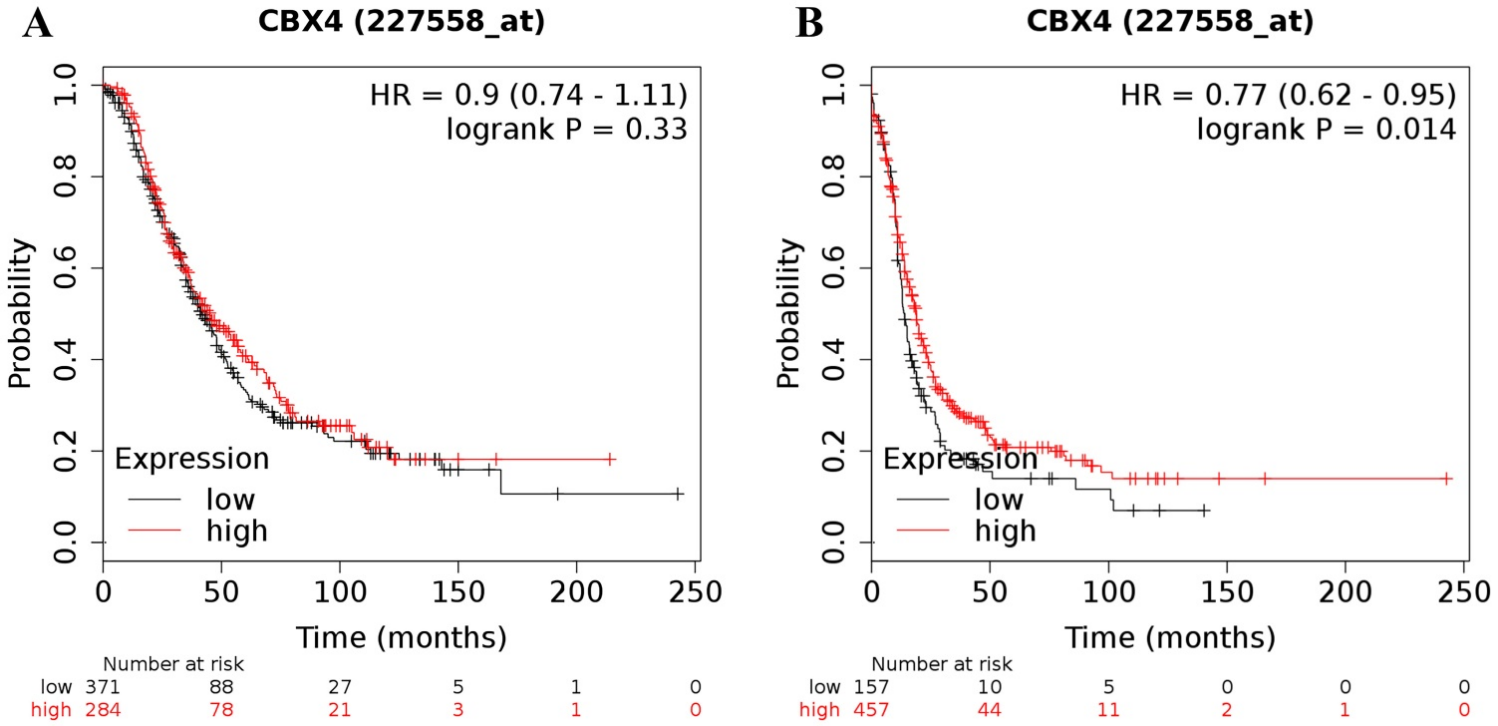
The prognostic value of CBX4 mRNA expression in ovarian cancer. Its Affymetrix ID is 227558_at. **(A)** OS curves are plotted for all ovarian cancer patients (n = 655). **(B)** PFS curves are plotted for all ovarian cancer patients (n = 614).

In contrast to the analyses for CBX1-3 in all ovarian cancer patients, analyses focusing on the expression of the other 5 CBX members revealed either inconsistent or irrelevant results for both OS and PFS in all patients with ovarian malignancy. For CBX4, its desired Affymetrix ID was 227558_at. Elevated expression of CBX4 mRNA was not related to OS for all ovarian cancer patients (HR = 0.90 (0.74 - 1.11), *P* = 0.33 (Figure 2A)). Nevertheless, elevated CBX4 mRNA expression was associated with a favorable PFS for all patients with ovarian carcinoma (HR = 0.77 (0.62 - 0.95), *P* = 0.014) (Figure 2B).

**Figure 3:**
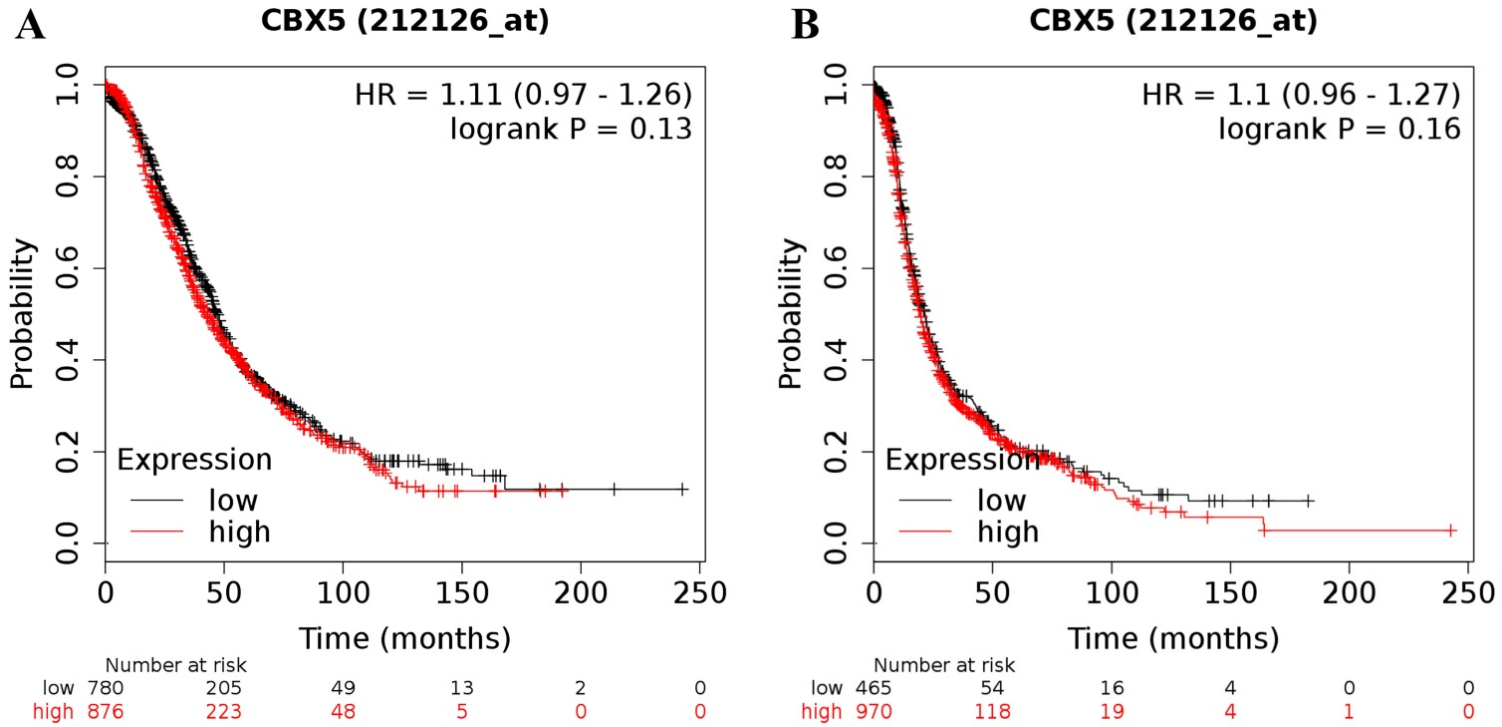
The prognostic value of CBX5 mRNA expression in ovarian cancer. Its Affymetrix ID is 212126_at.** (A)** OS curves are plotted for all ovarian cancer patients (n = 1,656). **(B)** PFS curves are plotted for all ovarian cancer patients (n = 1,435).

Increased expression of CBX5 (the Affymetrix ID is 212126_at) mRNA did not show any relation to the prognosis in all patients with ovarian cancer (for OS: HR = 1.11 (0.97 - 1.26), *P* = 0.13 (Figure 3A); for PFS: HR = 1.10 (0.96 - 1.27), *P* = 0.16 (Figure 3B)).

**Figure 4:**
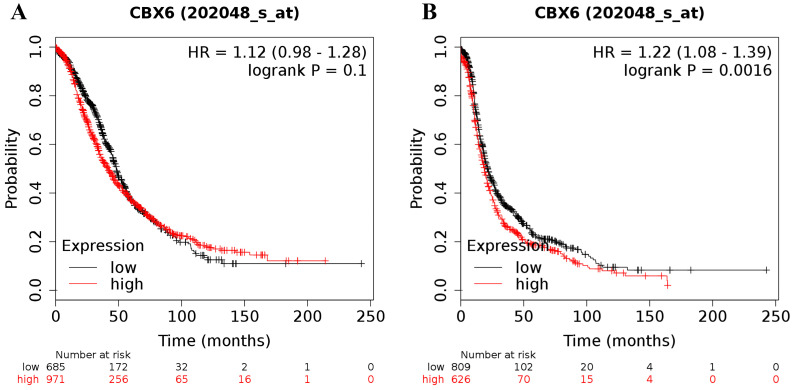
The prognostic value of CBX6 mRNA expression in ovarian cancer. Its Affymetrix ID is 202048_s_at. **(A)** OS curves are plotted for all ovarian cancer patients (n = 1,656). **(B)** PFS curves are plotted for all ovarian cancer patients (n = 1,435).

The desired Affymetrix ID of CBX6 was 202048_s_at. Upregulation of CBX6 mRNA had no effect on OS (HR = 1.12 (0.98 - 1.28), *P* = 0.1 (Figure 4A)) for all ovarian cancer patients, but was related to a worse PFS (HR = 1.22 (1.08 - 1.39), *P* = 0.0016 (Figure 4B)) for all ovarian cancer patients.

**Figure 5:**
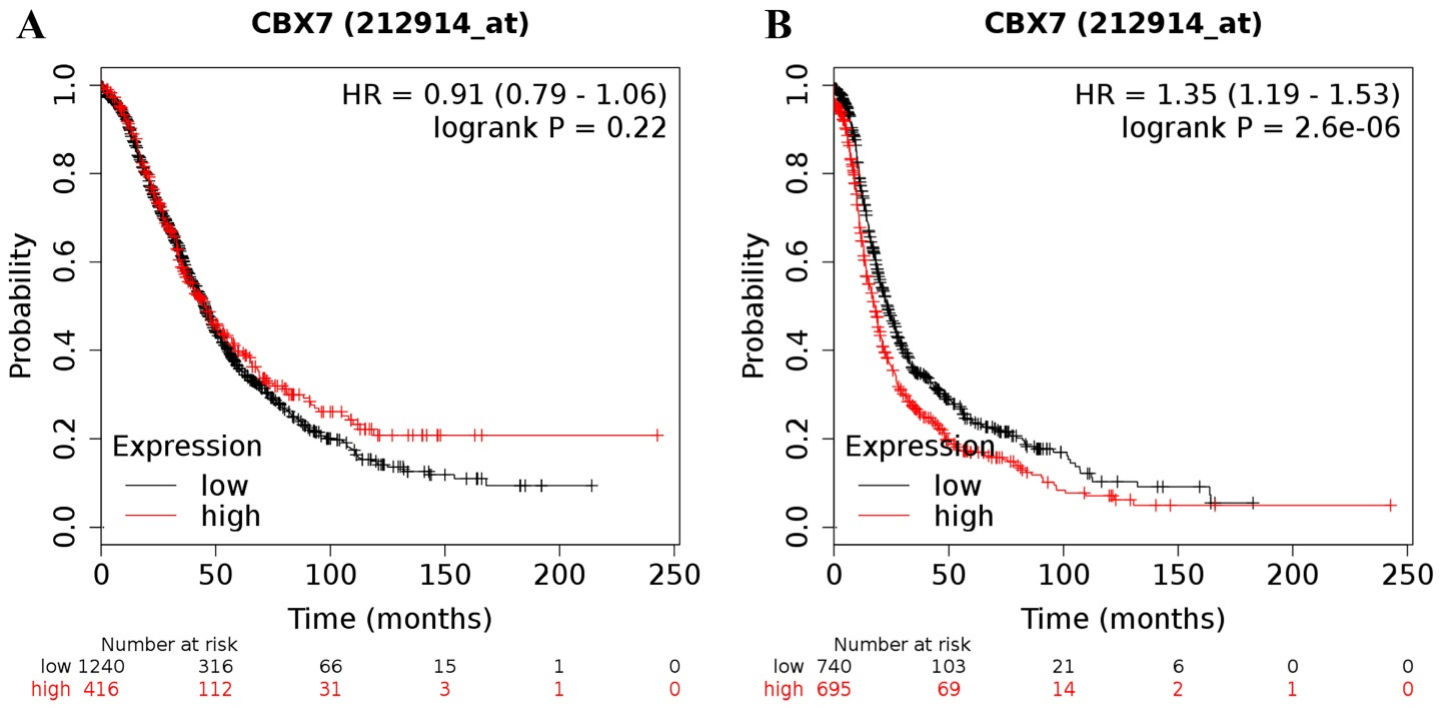
The prognostic value of CBX7 mRNA expression in ovarian cancer. Its Affymetrix ID is 212914_at. **(A)** OS curves are plotted for all ovarian cancer patients (n = 1,656). **(B)** PFS curves are plotted for all ovarian cancer patients (n = 1,435).

Moreover, the prognostic significance of CBX7 mRNA expression was determined in the database, with the desired Affymetrix ID for CBX7 of 212914_at. However, high expression of CBX7 mRNA was not related to OS (HR = 0.91 (0.79 - 1.06), *P* = 0.22 (Figure 5A)) for all ovarian cancer patients whereas was linked to a worse PFS (HR = 1.35 (1.19 - 1.53), *P* = 0.0000 (Figure 5B)) for all patients with ovarian carcinoma.

**Figure 6:**
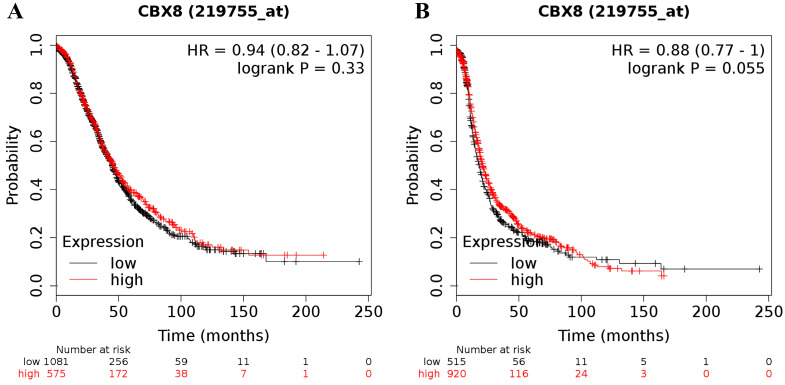
The prognostic value of CBX8 mRNA expression in ovarian cancer. Its Affymetrix ID is 219755_at. **(A)** OS curves are plotted for all ovarian cancer patients (n = 1,656).** (B)** PFS curves are plotted for all ovarian cancer patients (n = 1,435).

Finally, the CBX8 (Affymetrix ID was 219755_at) mRNA level was not related to the prognosis in all ovarian cancer patients (for OS: HR = 0.94 (0.82 - 1.07), *P* = 0.33 (Figure 6A); for PFS: HR = 0.88 (0.77 - 1.00), *P* = 0.055 (Figure 6B)).

Therefore, CBX1, CBX2 and CBX3 were selected for further analysis in diverse subgroups.

### Prognostic value of CBX1-3 mRNA expression in ovarian cancer of different histological subtypes

The prognostic roles of CBX1-3 mRNA expression in patients with ovarian malignant tumors were then investigated under different histological subtypes (Table 1). High expression of CBX1 mRNA was strongly related to poor OS (HR = 1.44 (1.20 - 1.73), *P* = 0.0001) and PFS (HR = 1.19 (1.01 - 1.39), *P* = 0.0341) for patients with serous ovarian malignancy. However, with regard to endometrioid ovarian cancer patients, there was no significant association with OS and PFS (for OS: HR = 4.41 (0.49 - 39.52), *P* = 0.1465, for PFS: HR = 0.47 (0.19 - 1.20), *P* = 0.1081).

Furthermore, elevated expression of CBX2 mRNA was shown to be involved in both poor OS (HR = 1.47 (1.11 - 1.95),* P* = 0.0066) and PFS (HR = 1.51 (1.18 - 1.93), *P* = 0.0010) for serous ovarian cancer patients. Nevertheless, there was no significant relationship between increased expression of CBX2 mRNA and OS in endometrial ovarian cancer patients (HR = 3.40×10^8^ (0 - Inf), *P* = 0.1665), while overexpression of CBX2 mRNA was linked to a better PFS (HR = 0.26 (0.08 - 0.83), *P* = 0.0149). In addition, serous ovarian cancer patients with elevated expression of CBX3 mRNA presented a significantly worse OS (HR = 1.25 (1.04 - 1.50), *P* = 0.0179) but a better PFS (HR = 0.84 (0.72 - 0.98), *P* = 0.0268). For endometrioid ovarian cancer patients, the CBX3 mRNA expression level was irrelevant to OS and PFS (HR = 0.35 (0.06 - 2.09), *P* = 0.2278, HR = 0.54 (0.21 - 1.36), *P* = 0.1817). Thus, the CBX1 and CBX2 mRNA levels in serous ovarian cancer patients were related to poor OS and PFS, and CBX3 mRNA was related to poor OS.

### Prognostic value of CBX1-3 mRNA expression in ovarian cancer patients with different pathological grades

Subsequently, the association between the mRNA expression of CBX1-3 and the survival outcome of ovarian cancer patients with diverse pathological grades was further evaluated (Table 2). The results revealed that highly expressed CBX1 mRNA had no significant influence on OS in ovarian cancer patients with grade I tumors. In patients with grade II and grade III diseases, upregulated expression of CBX1 mRNA was obviously correlated with poor OS. However, increased expression of CBX1 did not impact the PFS in all grades of ovarian carcinoma. For CBX2, an increased level of CBX2 mRNA was not involved in OS or PFS for all grades of ovarian cancer patients. For CBX3, improved expression of CBX3 mRNA was associated with unfavorable OS in women with grade II ovarian cancer. However, elevated CBX3 mRNA expression was irrelevant to OS both in patients with grade I and grade III ovarian cancer. Furthermore, elevated expression of CBX3 was not related to PFS in all grades of patients with ovarian cancer. Collectively, higher CBX1 mRNA expression was associated with worse OS in grade II and grade III patients with ovarian cancer. Moreover, increased CBX3 mRNA expression was linked to shorter OS in grade II patients.

### Prognostic value of CBX1-3 mRNA expression in ovarian cancer patients at different clinical stages

Next, the correlation between elevated expression of the CBX1-3 mRNAs and the prognosis of ovarian cancer patients at different clinical stages was investigated (Table 3). The results indicated that elevated levels of CBX1 mRNA were correlated with shorter OS but not PFS in patients at all stages. Elevated expression of CBX2 mRNA did not have any influence on prognosis in stage I and II ovarian cancer patients. However, upregulated CBX2 expression was significantly associated with a positive PFS in women with clinical stages III and IV ovarian cancer. Conversely, CBX2 expression at the transcriptional level was significantly related to unfavorable OS in stage III and IV ovarian cancer patients. In stage I and II ovarian cancer patients, the upregulated CBX3 mRNA level was a reliable factor for predicting poor PFS but not OS. Furthermore, upregulation of CBX3 expression was strongly correlated with poor OS in stage III and IV ovarian cancer patients. Interestingly, the results showed that high expression of CBX3 was significantly related to a positive PFS in women with clinical stages III and IV ovarian cancer. In general, increased CBX1 mRNA expression was obviously associated with poor OS in all stages of patients, while elevated expression of CBX2 and CBX3 was dramatically associated with poor OS in stages III and IV.

### Prognostic values of CBX1-3 mRNA expression in ovarian cancer patients treated with different chemotherapies

Table 4 summarizes the prognostic values of CBX1-3 mRNA expression in ovarian cancer patients administered different chemotherapies. In ovarian cancer patients who were treated with Taxol, platin, and Taxol+platin chemotherapy, increased CBX1 expression was involved in poorer OS and PFS. In particular, in patients who were treated with platinum, increased expression of CBX1 was strongly involved in poorer OS and PFS (HR = 1.37 (1.18 - 1.59), *P* = 0.0000, HR = 1.43 (1.24 - 1.64), *P* = 0.0000). Furthermore, there was no relationship between elevated CBX2 mRNA expression and OS in patients under treatment with Taxol, platin and Taxol+platin chemotherapy. Nevertheless, in patients treated with Taxol and Taxol+platin, high CBX2 mRNA expression was linked to an improved PFS. Additionally, an elevated expression level of CBX3 mRNA was associated with a shorter OS in patients who were treated with these three chemotherapeutic agents. Moreover, patients with high CBX3 mRNA expression also showed poor PFS after receiving platin chemotherapy.

### Clinical characteristics of ovarian cancer patients and control patients

From August 2017 to December 2018, the clinical characteristics of 18 patients with serous ovarian cancer and 18 patients with normal ovarian cancer were collected (there were 18 normal ovarian tissues obtained from bilateral oophorectomy due to unilateral ovarian benign lesions). The clinical characteristics of the patients such as age, region, histology, FIGO stage, grade, tumor size, type of therapy and marital status are summarized in Table 5.

### CBX1-3 proteins were strongly expressed in serous ovarian carcinoma tissues

**Figure 7:**
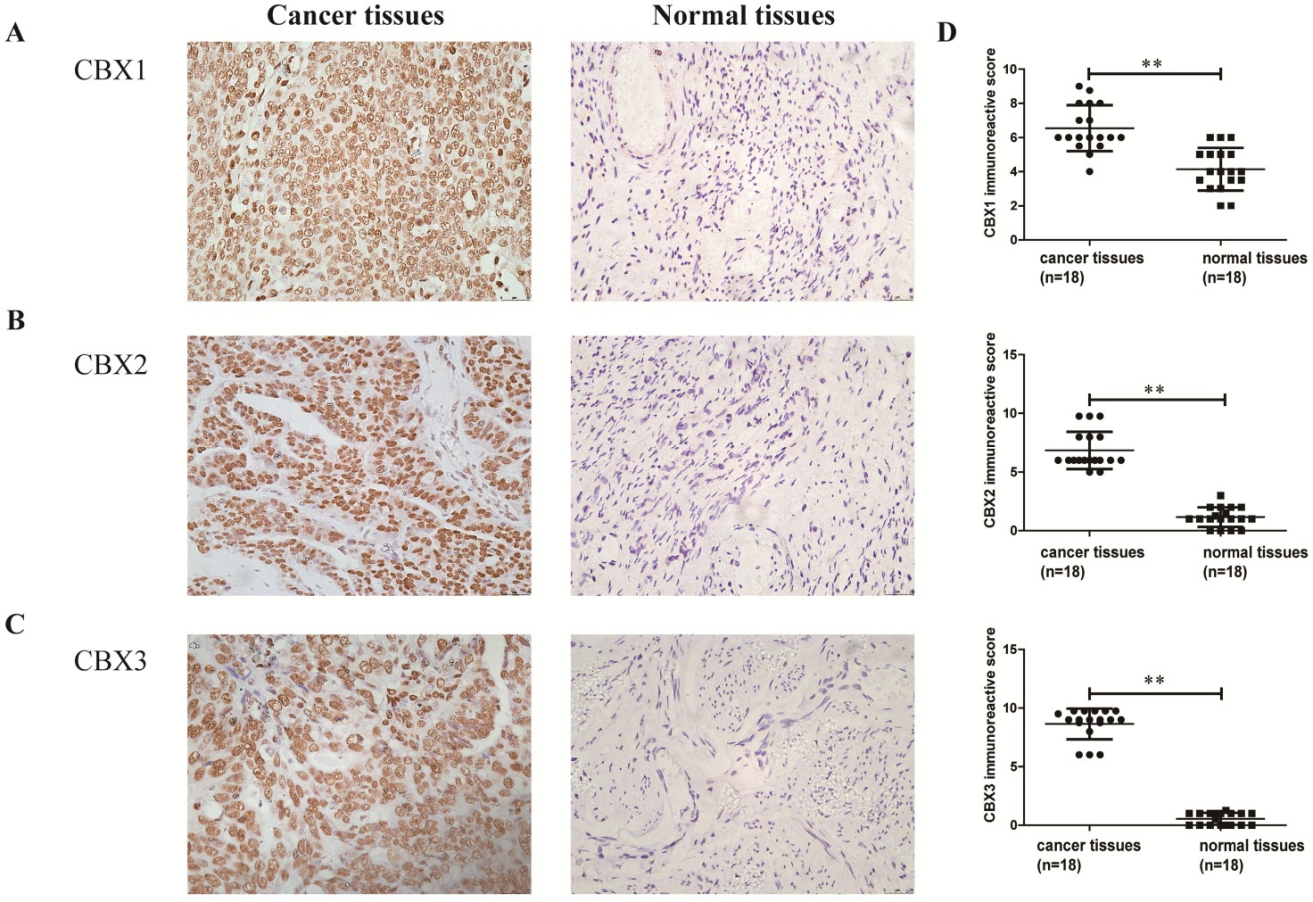
The expression levels of CBX1, CBX2 and CBX3 in serous ovarian cancer tissues and normal ovarian tissues. **(A-C)** The expression level of CBX1(A), CBX2(B) and CBX3(C) in serous ovarian cancer tissues and normal ovarian tissues (×400).** (D)** The intensity and frequency of staining in each tissue slide were scored and plotted as described in Materials and Methods. Values are expressed as the mean ± SD. *P < 0.05, **P < 0.01.

Finally, the expression levels of CBX1 and CBX2 at the transcriptional level in serous ovarian cancer patients were related to poor OS and PFS, and CBX3 was associated with poor OS. Thus women with serous ovarian carcinoma were selected as our research subjects. IHC staining was performed to identify whether the CBX1-3 proteins were overexpressed in serous ovarian cancer tissues compared with normal ovarian tissues. As shown in Figure 7, CBX1, CBX2 and CBX3 immunoreactivity was observed in the nucleus and yellowish brown cells were recognized as positive. CBX1 protein expression was obviously elevated in tumor specimens (6.54 ± 0.32) compared with normal ovarian tissues (4.14 ± 0.29, *P* < 0.01). Similar results were also obtained for CBX2 (tumor specimens: 6.85 ± 0.38; normal ovarian tissues: 1.15 ± 0.20, *P* < 0.01) and CBX3 (tumor specimens: 8.65 ± 0.31; normal ovarian tissues: 0.54 ± 0.12,* P* < 0.01) protein expression. In summary, our results showed that the CBX1, CBX2 and CBX3 proteins were overexpressed in serous ovarian carcinoma tissues compared with normal ovarian tissues.

## Discussion

Numerous studies have shown that CBX family isoforms are important regulators in tumorigenesis 15. To date, the expression level of individual CBX members and their prognostic roles in ovarian cancer have not been fully elucidated. In this study, the prognostic value of all eight CBX members was investigated among ovarian cancer patients by the KM plotter database. However, the results showed that the prognostic significance of CBX4-8 mRNA expression for predicting OS and PFS in all patients with ovarian cancer was inconsistent (CBX4, CBX6 and CBX7) or irrelevant (CBX5 and CBX8). Specifically, high expression levels of CBX1, CBX2 and CBX3 all predicted unfavorable OS and PFS in all ovarian cancer patients. Thus, CBX1-3, which had consistent results in the initial analysis, were selected for further investigation in our study. Then, the prognostic values of CBX1-3 mRNAs were further determined using the KM plotter database under various predefined subgroups including histological type, clinical and pathological grades, as well as different types of chemotherapeutic applications. In addition, it is widely accepted that genetic alterations are a common phenomenon in a diverse set of cancers and play a pivotal role in controlling cell cycle progression, apoptosis and cell growth 21. Subsequently, IHC analysis found that CBX1, CBX2 and CBX3 expression was significantly higher in ovarian cancer tissues than in the corresponding controls. Therefore, our results indicated that these three members may be reliable predictors of poor prognosis in ovarian cancer patients.

CBX1 and CBX3 belong to the HP1 group and play essential roles in the formation and maintenance of heterochromatin and gene regulation 22, 23. A study indicated that CBX1 and CBX3 have similar overlapping binding profiles between mice and humans 24. Additionally, CBX1 and CBX3 are involved in the transcriptional regulation of genes 25. One study indicated that CBX1 was a key regulator in regulating cell differentiation and proliferation, and silencing CBX1 inhibited prostate cancer cell proliferation 26. Yang et al. 12 also discovered that CBX1 protein expression was dramatically upregulated in HCC. It was suggested that HCC patients with elevated levels of CBX1 had shorter survival times and tumor recurrence times 12. Recently, Liang et al. 27 revealed that high expression of CBX1 might be predictive for poor clinical outcomes in breast cancer, especially in estrogen receptor-positive patients. Similarly, excessive expression of CBX1 was shown to be related to poor differentiation and unfavorable prognosis of breast cancer patients 28. Biologically, CBX1 knockdown inhibited prostate cancer cell proliferation by triggering cell cycle arrest in the G1 phase, indicating that CBX1 was positively related to cancer cell proliferation 29. In vitro data indicated that overexpressed CBX1 activated the Wnt/β-catenin signaling pathway through the transcription factor HMGA2, thus promoting HCC cell growth and migration 12. Knockdown of CBX1 or inhibition of β-catenin markedly reduced the CBX1-mediated cell growth in HCC 12.

The prognostic value of CBX1 in ovarian carcinoma and the expression of CBX1 at the transcriptional level and translational level in ovarian cancer tissues have not been further investigated. In our research, we found that upregulation of CBX1 expression was obviously associated with worse prognosis in all patients with ovarian carcinoma, especially for those with the serous histological type, grade II, grade III, and all stages and for those treated with Taxol, platin, or Taxol+platin. Especially in patients receiving platinum treatment, increased expression of CBX1 was strongly related to poorer OS and PFS. Platinum and Taxol are relatively effective first-line therapeutic agents for patients with ovarian cancer, but chemotherapy resistance has led to poor outcomes 30. Another challenge is the lack of biomarkers for ovarian cancer patients to predict the chemotherapeutic treatment response 31. These findings indicated that CBX1 can be used as a biomarker for adverse outcomes and poor chemotherapeutic response in patients with ovarian cancer. IHC staining analysis indicated that CBX1 expression in ovarian carcinoma specimens was obviously higher than that in normal ovarian tissues. Taken together, our results showed that CBX1 may be a vital regulator in the carcinogenesis of ovarian cancer and acted as a promising candidate for predicting prognosis.

Some studies have indicated that CBX3 expression is upregulated in many human cancers, such as cervical cancer, breast cancer and lung cancer 27, 32-34. Suppression of CBX3 expression had an inhibitory effect on cervical cancer cell growth, demonstrating that CBX3 might be an effective therapeutic target for cervical cancer 33. Consistently, Liu et al. 11 indicated that CBX3 expression was increased in colorectal tumors and triggered cell proliferation by the miR-30a/HP1γ/p21 regulatory axis, which suggested that the CBX3 protein was a carcinogenic molecule in the development of human colorectal cancer 11. These findings paralleled the IHC results in our research showing that CBX3 expression was generally upregulated in most ovarian carcinoma tissues compared with normal ovarian tissues. In prostate cancer, CBX3 protein expression was increased, and Cox survival analysis showed that it was an independent prognostic marker. Additionally, the correlation between CBX3 protein expression and clinically unfavorable outcomes was stronger than the correlation in prostate cancer 35. Elevated CBX3 mRNA levels were involved in poor prognosis in lung adenocarcinoma patients 34. This growing evidence identified CBX3 as an essential molecule in the tumorigenesis and treatment of human cancers. Nevertheless, the prognostic value of CBX3 in ovarian carcinoma is unclear. Upregulation of CBX3 mRNA was obviously involved in worse prognosis for all ovarian cancer patients and in patients who were treated with Taxol, platin, and Taxol+platin chemotherapy. A growing number of studies have verified that CBX3 has an essential impact on prognosis and that CBX3 may participate in the carcinogenesis of ovarian cancer.

In terms of structure and function, CBX2 is one of the essential components of PRC1, which is different from CBX1 and CBX3. Of the eight CBX families in humans, only CBX2 has a strong preference for histone H3 lysine-27trimethylation (H3K27me3), suggesting that CBX2 may be a functional ortholog of Drosophila HP1 in humans 36. Another article found that CBX2 is also the only CBX protein with a DNA binding domain 37, which is associated with modulating transcription of target genes and binding to other PRC1 components and is involved in the development of human cancer 38. A meta-analysis of CBX2 gene transcription showed that CBX2 mRNA expression was higher in many human cancer tissues than in normal tissues 39. Elevated expression of CBX2 at both the transcriptional and translational levels was observed in aggressive prostate cancer 13. Notably, previous studies showed that in primary high-grade serous ovarian carcinoma, CBX2 protein expression was obviously increased compared with that in normal cancer tissues 40. In HCC tissues, strong increases in higher levels of CBX2 expression at the mRNA and protein levels were found, and CBX2 expression at the transcriptional level was positively correlated with cancer stages and grades 41. In this study, IHC staining demonstrated that CBX2 expression was obviously elevated at the translational level in serous ovarian cancer tissues compared with normal ovarian tissues, which was similar to a previous finding 40. Recently, Zheng et al. 42 found a link between increased CBX2 expression and worse prognosis of breast cancer patients. Our findings demonstrated that high expression of CBX2 at the transcriptional level was related to an adverse prognosis in all patients, especially in patients with serous ovarian malignancy. Therefore, CBX2 might be a potential unfavorable prognostic candidate for ovarian cancer.

## Conclusions

In summary, our study reveals that CBX1-3 are prognostic candidates for predicting unfavorable clinical outcomes in ovarian cancer patients. In addition, it has been observed that CBX1-3 proteins are expressed at higher levels in serous ovarian cancer tissues than in normal ovarian tissues. These results provide evidence to support the view that CBX1-3 can become new prognostic candidates and be developed as drug therapeutic targets for ovarian cancer.

## Figures and Tables

**Table 1:** Correlation of CBX members mRNA expression level with OS and PFS in different histological subtypes of ovarian cancer patients

CBX	Histologic subtypes	OS			PFS		
		Cases	HR (95% CI)	*P* value	Cases	HR (95% CI)	*P* value
CBX1	serous	1207	1.44 (1.20 - 1.73)	0.0001*	1104	1.19 (1.01 - 1.39)	0.0341*
	endometrioid	37	4.41 (0.49 - 39.52)	0.1465	51	0.47 (0.19 - 1.20)	0.1081
CBX2	serous	523	1.47 (1.11 - 1.95)	0.0066*	483	1.51 (1.18 - 1.93)	0.0010*
	endometrioid	30	3.40×10^8^ (0 - Inf)	0.1665	44	0.26 (0.08 - 0.83)	0.0149*
CBX3	serous	1207	1.25 (1.04 - 1.50)	0.0179*	1104	0.84 (0.72 - 0.98)	0.0268*
	endometrioid	37	0.35 (0.06 - 2.09)	0.2278	51	0.54 (0.21 - 1.36)	0.1817
							

*P <0.05.

**Table 2:** Correlation of CBX members mRNA expression level with OS and PFS in different pathological grades of ovarian cancer patients

CBX	Grades	OS			PFS		
		Cases	HR (95% CI)	*P* value	Cases	HR (95% CI)	*P* value
CBX1	I	56	2.00 (0.77 - 5.21)	0.1496	37	3.67 (0.81 - 16.60)	0.0710
	II	324	1.47 (1.05 - 2.05)	0.0240*	256	1.30 (0.93 - 1.82)	0.1182
	III	1015	1.41 (1.16 - 1.71)	0.0004*	837	1.11 (0.94 - 1.31)	0.2144
CBX2	I	41	1.56 (0.43 - 5.64)	0.4961	28	2.75 (0.57 - 13.26)	0.1881
	II	162	1.44 (0.92 - 2.24)	0.1103	161	1.21 (0.82 - 1.78)	0.3348
	III	392	1.27 (0.97 - 1.66)	0.0803	315	0.79 (0.60 - 1.04)	0.0896
CBX3	I	56	2.34 (0.67 - 8.20)	0.1711	37	1.82 (0.60 - 5.59)	0.2855
	II	324	1.38 (1.01 - 1.88)	0.0434*	256	0.78 (0.56 - 1.10)	0.1532
	III	1015	1.19 (0.98 - 1.44)	0.0753	837	0.86 (0.72 - 1.03)	0.0983

*P <0.05.

**Table 3:** Correlation of CBX members mRNA expression level with OS and PFS in different clinical stages of ovarian cancer patients

CBX	Clinical stages	OS			PFS		
		Cases	HR(95% CI)	*P* value	Cases	HR (95% CI)	*P* value
CBX1	I+II	135	4.65 (1.10 - 19.68)	0.0215*	163	1.69 (0.95 - 2.99)	0.0712
	III+IV	1220	1.39 (1.17 - 1.65)	0.0002*	1081	1.07 (0.92 - 1.25)	0.3714
CBX2	I+II	83	2.47 (0.88 - 6.89)	0.0753	115	1.77 (0.85 - 3.69)	0.1212
	III+IV	487	1.33 (1.01 - 1.76)	0.0399*	494	0.78 (0.63 - 0.96)	0.0213*
CBX3	I+II	135	1.81 (0.68 - 4.82)	0.2265	163	2.33 (1.30 - 4.17)	0.0035*
	III+IV	1220	1.25 (1.06 - 1.49)	0.0089*	1081	0.82 (0.70 - 0.95)	0.0101*

*P < 0.05.

**Table 4:** Correlation of CBX members mRNA expression level with OS and PFS in different chemotherapy of ovarian cancer patients

CBX	chemotherapy	OS			PFS		
		Cases	HR (95% CI)	*P* value	Cases	HR (95% CI)	*P* value
CBX1	Platin	1409	1.37 (1.18 - 1.59)	0.0000*	1259	1.43 (1.24 - 1.64)	0.0000*
	Taxol	793	1.44 (1.18 - 1.77)	0.0004*	715	1.23 (1.02 - 1.49)	0.0271*
	Taxol+Platin	776	1.45 (1.18 - 1.78)	0.0003*	698	1.24 (1.03 - 1.50)	0.0262*
CBX2	Platin	478	1.28 (0.99 - 1.66)	0.0570	502	0.82 (0.66 - 1.01)	0.0669
	Taxol	357	0.84 (0.62 - 1.16)	0.2938	381	0.75 (0.59 - 0.95)	0.0167*
	Taxol+Platin	356	0.84 (0.61 - 1.15)	0.2850	380	0.75 (0.59 - 0.95)	0.0183*
CBX3	Platin	1409	1.23 (1.06 - 1.42)	0.0068*	1259	1.26 (1.09 - 1.46)	0.0022*
	Taxol	793	1.27 (1.05 - 1.55)	0.0161*	715	1.11 (0.93 - 1.32)	0.2399
	Taxol+Platin	776	1.30 (1.06 - 1.59)	0.0112*	698	1.13 (0.94 - 1.34)	0.1845

*P <0.05.

**Table 5:** Clinical characteristics of ovarian cancer patients and control patients

Variables	Patients with ovarian cancer (N=18)	Patients with normal ovarian tissues (N=18)
Median age (years)	50 (range 34-65)	56 (range 45-63)
**Histology, n (%)**		
Serous	18 (100.0)	─
Clear cell	0 (0)	─
Endometrioid	0 (0)	─
Mucinous	0 (0)	─
**FIGO stage, n (%)**		
I+II	8 (44.4)	─
III+IV	10 (55.6)	─
**Grade, n (%)**		
low	8 (44.4)	─
high	10 (55.6)	─
**Tumor size (cm)^ a^, n (%)**		
≤4	1 (5.6)	─
4-7	4 (22.2)	─
7-10	6 (33.3)	─
≥10	5 (27.8)	─
**Type of therapy, n (%)**		
Surgery + Chemotherapy	18 (100.0)	─
Surgery + Radiation	0 (0)	─
Surgery +Radiation+ Chemotherapy	0 (0)	─
**Marital status, n (%)**		
Married	18 (100.0)	18 (100.0)
Other	0 (0)	0 (0)

a: The sum of the numbers for the variable is less than the total due to missing values.

## Abbreviations

BSA: bovine serum albumin
CBX: chromobox
DAB: 3,3'-diaminobenzidine
HCC: hepatocellular carcinoma
HP1: heterochromatin protein 1
H3K27me3: Histone H3 lysine-27 tri-methylation
IHC: immunohistochemistry
OS: overall survival
PFS: progression-free survival
PRC1: polycomb repressive complexes 1
TCGA: The Cancer Genome Atlas
